# Accurate Spirometry with Integrated Barometric Sensors in Face-Worn Garments

**DOI:** 10.3390/s20154234

**Published:** 2020-07-29

**Authors:** Bo Zhou, Alejandro Baucells Costa, Paul Lukowicz

**Affiliations:** 1 Research Group Embedded Intelligence, German Research Center for Artificial Intelligence, 67663 Kaiserslautern, Germany; Alejandro.Baucells_Costa@dfki.de (A.B.C.); paul.lukowicz@dfki.de (P.L.); 2 Department of Computer Science, University of Kaiserslautern, 67663 Kaiserslautern, Germany

**Keywords:** wearable healthcare, biomedical sensing, spirometry, pulmonary signals, barometer

## Abstract

Cardiorespiratory (CR) signals are crucial vital signs for fitness condition tracking, medical diagnosis, and athlete performance evaluation. Monitoring such signals in real-life settings is among the most widespread applications of wearable computing. We investigate how miniaturized barometers can be used to perform accurate spirometry in a wearable system that is built on off-the-shelf training masks often used by athletes as a training aid. We perform an evaluation where differential barometric pressure sensors are compared concurrently with a digital spirometer, during an experimental setting of clinical forced vital capacity (FVC) test procedures with 20 participants. The relationship between the two instruments is derived by mathematical modeling first, then by various regression methods from experiment data. The results show that the error of FVC vital values between the two instruments can be as low as 2∼3%. Beyond clinical tests, the method can also measure continuous tidal breathing air volumes with a 1∼3% error margin. Overall, we conclude that barometers with millimeter footprints embedded in face mask apparel can perform similarly to a digital spirometer to monitor breathing airflow and volume in pulmonary function tests.

## 1. Introduction

Our cardio-respiratory (CR) system is the most important physiological system, as it is responsible for oxygen delivery to the entire body. In recent years, many wearable approaches have emerged to monitor CR vital signals both in the academic research field and the consumer electronics sector [1].

Non-invasive CR evaluation includes lung function tests (pulmonary function tests (PFTs)) and heart tests. PFTs include spirometry, which measures the physical aspects of the lung’s functionality such as the lung volume and rate of flow. PFTs also concern the gas chemical composition, which usually requires gas chromatography and mass spectrometry [2,3]. Heart tests usually include the well-known electrocardiograph (ECG), blood flow tests (pulse-oximetry), and blood pressure tests. These vital signs are commonly tested during patients’ diagnosis and athlete’s performance evaluations. Pulmonary tests especially play an important role in the diagnosis and evaluating the progression of respiratory and pulmonary diseases, as well as post recovery after other medical procedures (e.g., post operation) [4,5,6].

Wearable healthcare devices [7] provide the possibility to monitor vital signals and health-related activities in people’s daily lives, which benefits both peoples awareness towards their own health and the healthcare providers’ understanding of the patients’ progress. Personal wearable devices can also reduce the chance of the cross-infection of respiratory diseases, which may be a concern of shared medical devices [8]. This becomes more relevant as the pulmonary function is a major indicator for such diseases. Heart-related parameters, in particular heart rate monitoring during physical exercise, is among the most popular and successful applications of wearable systems. As far as PFT is concerned, research has focused on wearable breathing frequency monitoring, and some systems suitable for long-term real-life deployment have been demonstrated (as explained in Section 2). While breathing frequency is a relevant factor in PFTs, without information on the related breathing volume, it has limited value only. Many studies have shown the two factors to be independent of each other. For example, the breathing frequency-to-volume ratio can vary and be the indicator of signs of fatigue, external stimulus, etc. [9,10,11,12]. To date, breathing volume measurement is mostly restricted to constrained laboratory settings due to the form factor of existing spirometers. To measure the airflow, spirometers typically require directing all the air flow from the patient to a sensing element (as explained in Section 2). Such measurement modalities prevent miniaturizing spirometers, because if the vent cross-section area is too small, the user’s breathing will be restricted. This is what our work aims to address.

### 1.1. Novelty and Contribution

**Figure 1:**
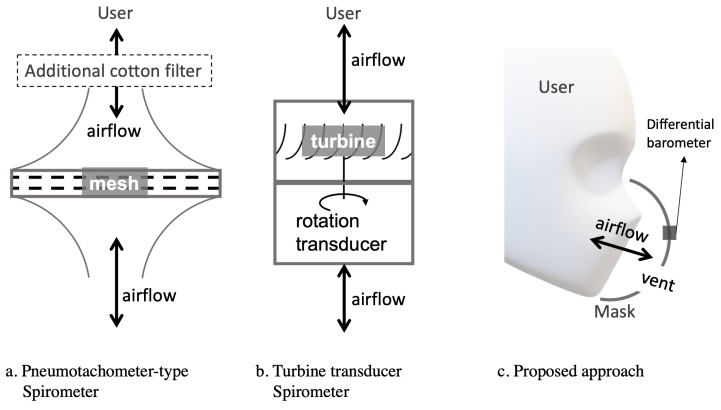
Sensing modality comparisons between current typical mobile spirometers and our approach. (**a**) Pneumotachometer-type and (**b**) turbine-type spirometers all require mechanical parts that cover the entire airway; (**c**) our approach only requires millimeter-scale barometric sensors on a normal facial mask and does not require the entire airway.

Our major novelties and contributions are:

- We demonstrate the possibility of performing accurate transient breathing volume measurement in a wearable garment in the form of a sports mask, as opposed to hand-held novel spirometers, which mostly require a specific structure with a breathing tube, as reviewed in Section 2.2.
- Our approach uses only off-the-shelf components, without any proprietary sensors or custom mechanical designs.
- The only sensing element needed is a pair of low-cost (three Euros) miniaturized (2.5 millimeters) barometric pressure sensors that are already widely available thanks to the personal mobile devices and drone industry.
- The approach is made possible by shifting the measurement modality, from directly placing sensors in the airflow duct to elaborating the pressure difference of the inside and outside of the face mask compartment, as shown in Figure 1.

We evaluate the approach with an off-the-shelf clinical spirometer in an experiment consisting of 20 participants performing in total 200 forced vital capacity (FVC) tests and additional normal breathing. A physical model is constructed to theoretically explain the relationship between the airflow and the barometric pressure in our setting. Then, regression methods are performed to derive the relationship between the two instruments from the evaluation experiment data. Through our approach, clinical pulmonary function parameters such as forced effective volume in one second (FEV_1), etc., are calculated both from the clinical spirometer and our integrated barometric sensor approach. We compare both the transient breathing airflow and volume, as well as the FVC vital parameters. We also compare our approach to multiple state-of-the-art novel hand-held spirometer research results, as well as the medical society requirements for spirometry. We conclude that our approach is sufficient to perform accurate pulmonary function tests on a similar level as a clinical digital spirometer.

### 1.2. Paper Structure

In Section 1, the motivation and contribution of this work are stated. The relevant background and state-of-the-art are introduced in Section 2. In Section 3, we describe the apparatus in this study, including the instrumented sports mask and the calibration setting where our approach is connected with a spirometer to share the same airflow. In Section 4, a mathematical model is derived for our smart mask setting to explain the relationship between the air pressure and airflow, which are measured by the two different instruments in the evaluation apparatus. The evaluation procedure is described in Section 5. Section 6 explains the algorithms we developed to validate our approach. The results of the evaluation are then presented in Section 7. Section 8 concludes the entire paper with the major findings and outlook.

## 2. Background and State-of-the-Art

### 2.1. Pulmonary Function Tests

The PFT Forum (www.pftforum.com.) offers a comprehensive archive of the history in medical and sport respiratory evaluation. PFTs usually require a patient to fully inhale and then fully exhale with maximum force. The important factors those tests seek to determine include the air capacity of lungs, as well as the transient flow rate in different stages of the total capacity.

**Figure 2:**
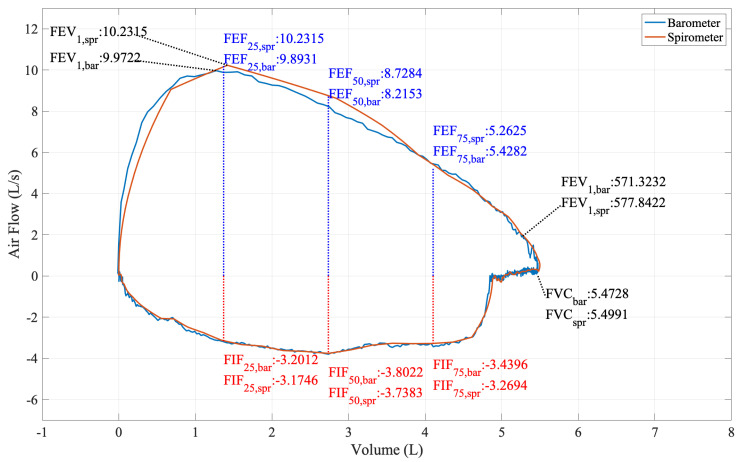
Flow-volume loop of a FVC maneuver from Participant 1.

**Figure 3:**
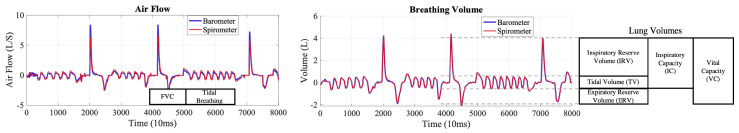
The airflow and air volume of three consecutive FVC maneuvers, with normal tidal breathing in between. The spirometer’s signals are the original measurement; the barometer’s values are predicted with the inclusive-poly5 model.

The most common clinical pulmonary function test is the forced vital capacity test [13]. The FVC maneuver consists of three steps: maximal inspiration, explosive exhalation, and continued complete exhalation. From the test, typically, a flow-volume loop is generated (e.g., the red curve in Figure 2). From the FVC test and the flow-volume loop, further essential parameters are calculated that reflect the subject’s lung condition, such as forced vital capacity (FVC), forced expiratory volume in one second (FEV1), forced peak expiratory flow (PEF), forced expiratory flow at x% of FVC (FEFx), forced inspiratory flow at x% of FVC (FIFx), etc. Other pulmonary function parameters such as tidal volume (TV), inspiratory reserve volume (IRV), expiratory reserve volume (ERV), and inspiratory capacity (IC) can be derived from the combination of FVC maneuvers and normal tidal breathing, as shown in Figure 3. However, some pulmonary function parameters including the residual volume (RV), functional residual capacity (FRC), total lung capacity (TLC), etc., cannot be directly measured through clinical procedures using spirometry alone [14,15], with which we will not be further concerned in this study.

### 2.2. Spirometry Devices

The earliest spirometers are completely mechanical devices that require visual readings. Specifically, the incentive-type spirometers [16] are still being prescribed to patients to perform regular PFTs at home. The mechanical (including incentive-type) spirometers usually have a disk or piston inside an air chamber. The more air the patient exhales, the higher the disk or piston would move in the chamber. Apart from measuring the lung capacity, such PFT procedures can also help the patients to practice deep breathing and improve their pulmonary functions, as some randomized controlled trials suggest [16,17].

However, the mechanical spirometers usually only provide information about the total lung capacity, not the transient flow rate, due to the limitation of the visual reading. Thus, to inspect other pulmonary information, such as FEV1, PEF, FEFx, FIFx, etc., digital spirometers that can record continuous flow rate information during pulmonary function tests were developed. Earlier studies typically used complex and bulky systems to measure expiratory airflow such as the Otis-McKerrow valve-Fleisch pneumotachograph–Validyne manometer setting in [3]. Currently, most commonly used devices for respiratory evaluation are hand-held or stationary devices such as the pneumotachometer-type spirometer shown in Figure 1a or the turbine-type spirometer [18] in Figure 1b.

They are typically used while the patients or subjects are seated steadily. To be able to use spirometers in sports exercises with stationary machines such as a treadmill or an ergometer, a mouthpiece and tubing are needed to connect from the person’s airway to the stationary devices, such as the professional CR analyzer Metalyzer${}^{®}$ 3B by Cortex Medical or the PRE-201${}^{®}$ produced by Piston Medical, which can also analyze gas composition. However, with a mouthpiece, the user’s nose needs to be closed with a clip so that air will only exchange through the mouth. As an alternative, a complete face cover with tubing can also clinically replace a mouthpiece to direct airflow into the spirometers [19,20].

The overall trend of device evolution is apparently towards smaller and less expensive spirometer devices, with the main arguments that patients can perform tests on their own in their daily life, with the emerging small devices such as AioCare${}^{®}$ and MIR Smart One${}^{®}$. Medical research shows that some hand-held spirometers are suitable for clinical trials [21,22], yet the turbine type in particular underestimates key PFT values. Cortex Medical also has a wearable system Metamax${}^{®}$ 3B that consists of a measurement mask and chest or back carried additional hardware in a thin bag, overall weighing 580 g. Yet, among all our findings, only the Metamax${}^{®}$ 3B is oriented toward untethered sports activities.

Apart from the medical equipment mentioned above, much effort has been devoted toward novel respiration rate monitoring methods in the academic field [23]. A novel spirometer using a specially-designed micro-electro-mechanical system (MEMS) airflow sensor was demonstrated in [24], reporting 3∼5% accuracy in FVC and FEFx tests. In [25], another specially-made MEMS airflow sensor was produced and compared with a pneumotach spirometer, resulting in a coefficient of determination of 0.986 (one is perfect correlation) in airflow values without PFT parameters such as FVC or FEFx. However, both MEMS elements require a dedicated construct of several centimeters, and all the airflow needs to be directed into the construct. An optical fiber-based airflow sensor was evaluated for pulmonary function tests purposes in [26], reporting 20% error rates for FVC values compared with a pneumotachograph spirometer. An open architecture reconfigurable respiration monitor was proposed in [27] using an off-the-shelf airflow sensor and other gas composite sensors to perform PFTs, with a Pearson correlation of 0.94. In [28], a wireless hand-held pneumotachograph spirometer was developed, showing a 3% error for FVC and a 10% error for PEF values.

Yet, all of the above-mentioned novel spirometer research stays with the conventional spirometer structural topology: a flow-rate transducer placed inside a breathing tube as a handheld device, with the user breathing through the tube structure. There are also various research works that depart from the conventional spirometer structure, elaborating wearable and pervasive sensing methods that do not directly measure the airflow, to monitor respiratory activities.

### 2.3. Respiration Monitoring in Wearable and Pervasive Research

A significant merit of wearable and pervasive sensing methods is that they enable everyday garments or objects with novel sensing capabilities to detect the users’ activities or assist their daily life; while keeping the garments’ or objects’ originally intended functionalities, or at least the novel garments are unobtrusive or not distracting for the users during their daily activities.

In works such as [29,30,31], pneumatics-based systems are used to detect respiration and heart beat movements by pressure sensors in the air cells under sleeping mattresses. The pneumatics systems detect the body weight shifts caused by respiratory and heart beat activities. Wearable approaches have also been investigated. In [32,33], capacitive sensing was deployed at the subjects’ chest area to detect breathing and pulse signals. As the girths of chest and abdomen show a clear correlation with breathing activities [34], many works have used wearable stretch or bend sensors around the chest to detect breathing cycles [35,36,37,38,39]. However, none of the works mentioned above have investigated a quantifiable correlation between the chest girth and the absolute breathing volume. In fact, as pointed out in [40], there is little correlation between the vital lung capacity and the chest or trunk girth. In [41], a low-power sound sensing system placed at the wearer’s neck was developed to detect respiratory events such as asthmatic wheezing.

Barometers measure the air pressure and are integrated in many wearable and mobile devices and drones currently, since the atmospheric pressure difference can be used to estimate the change of the altitude elevation [42,43,44]. In [45,46], integrated barometers were placed inside face masks to monitor breathing activities without distracting the wearer, from which the intensity was classified as “normal breathing” and “deep breathing” through machine learning approaches. To the best of our knowledge, there is currently no study on how the precise airflow can be measured by miniaturized sensors in the chip form factor, embedded in off-the-shelve wearable apparel to reach the level of clinical pulmonary function tests.

## 3. Hardware

In this section, we first describe the data acquisition hardware prototype with the embedded barometers in face mask apparel. To convert the barometric pressure to spirometer airflow, we connected our prototype to an off-the-shelf digital spirometer to share the same airway (the mask-to-spirometer (M2S) setup). The algorithms and evaluations from Section 4 to Section 7 are based the M2S setup. To verify that the airway connection cone and spirometer placement needed for the M2S setup do not have a significant impact on the spirometry result, we replaced our prototype with another identical spirometer (the spirometer-to-spirometer (S2S) setup) and performed preliminary validations with the S2S setup.

### 3.1. Embedded Barometer and Electronics Hardware

We compared two embedded barometric sensors: BMP280 and BMP388, both manufactured by Bosch Sensortec${}^{®}$. They measure atmospheric pressure and temperature. BMP280 has a footprint of 2.5 × 2.5 mm and a height of 0.95 mm, a 182Hz sampling rate, and a ±1 hPa absolute pressure accuracy between 300 and 1100hPa, according to the datasheet. BMP388 is an improved and newer version of BMP280. It has a smaller footprint of 2 × 2 mm and a height of 0.75 mm, a higher 200 Hz sampling rate, and a ±1 hPa absolute pressure accuracy. The cost was around 2EUR for BMP280 and 3EUR for BMP388 at the time of writing.

The barometers were placed on the inside and outside as differential pairs of a mask with a rubber face seal (Training Mask${}^{®}$ 3.0). The mask has a valve system to adjust the airflow resistance. The valves were completely removed in our prototype so that the wearer could breath freely. Volunteers who tried the prototype reported no noticeable resistance when breathing with the mask.

In initial trials, we observed an offset in the pressure values from different sensors in the same room. Therefore, we devised a differential barometer setup, with one barometer inside and one outside the mask. Thus, the difference between these two sensors’ pressure values is the offset plus the true pressure difference.

**Figure 4:**
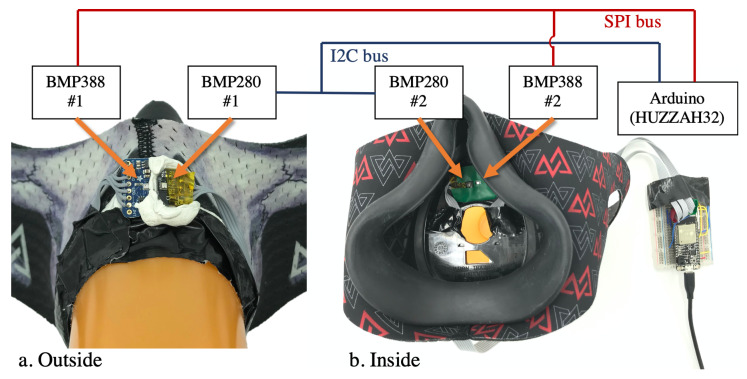
The hardware and communication structure during the experiment: outside view (**a**) and inside view (**b**) of the facemask.

Figure 4 shows the electronics hardware in our study. A pair of BME280 and a pair of BME388 sensor modules (breakout boards) were connected to one Arduino${}^{®}$ board (HUZZAH32) with an I2C bus (for BMP280) and an SPI bus (for BMP388). This configuration ensured that the four sensors were synchronized on the hardware level. The data of all four sensors were read out together at 10ms intervals (100 Hz) and then downsampled by two-sample averaging to 50 Hz to match the digital spirometer’s sampling rate. The data were then sent to a computer (Dell${}^{®}$ XPS9650) via a USB cable with the standard serial port protocol.

**Figure 5:**
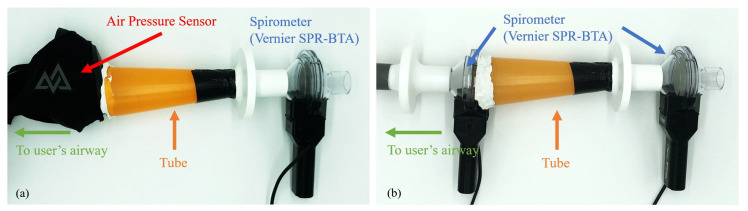
Calibration setup of the BME280 sensor for spirometry: (**a**) M2S setup, (**b**) S2S setup.

A medically-certified digital pneumotachometer-type spirometer was used (Vernier${}^{®}$ SPR-BTA) as our reference, as shown in Figure 5. It measures the airflow with a 7cm diameter round disk of fine mesh. The data were sent to the same computer via a proprietary USB adapter (Go!Link) and saved by the data logger software from Vernier${}^{®}$.

### 3.2. Mask-to-Spirometer Calibration Setup

In this study, we used a tube to connect the airflow between the instrumented mask and the spirometer so that both devices shared the same airflow, as shown in Figure 5a. This allowed us to calibrate the air pressure inside the mask chamber measured by the BME280 sensor with the flow rate measured by the spirometer. The tube’s narrower end was sealed with the spirometer’s inlet with duct tape (tesa${}^{®}$ extra Power Universal), and the wider end was sealed with the mask with an excessive amount of solid caoutchouc adhesive (UHU${}^{®}$ Patafix). To test the air-tightness, prior to the recording, the participants were asked to check that they could neither breath in nor out with the spirometer’s outlet sealed with their palm. During the recording, the participants held the spirometer and pressed it against the mask to ensure air-tightness.

Three people in their 20s participated in the calibration recording. The participants were chosen so that they had different body sizes to introduce more variation (Participant 1: male, 185 cm; Participant 2: female, 163 cm; Participant 3: male, 196cm). According to various studies such as [47,48], body size has strong direct correlations with the volume, control, and pattern of breathing. Each participant breathed in a normal, but focused manner with the setup for approximately three minutes. The spirometer had a stable sampling rate of 50 Hz. Our system also read the barometers’ value at the same 50 Hz, although the embedded barometers were capable of sampling rates of up to 200 Hz. Naturally, the data from different devices did not exactly have the same timestamps. The two devices’ data used the clock from the same receiver computer; therefore, the data shared the same time scale. They were interpolated with the linear method to the same time granularity of 10 ms, which was decided to be half of the devices’ sampling period according to the Nyquist–Shannon sampling theorem.

### 3.3. Spirometer-to-Spirometer Reference

Since the tube was customized just for this study, it was unknown whether it was introducing any air friction or disturbance to the airflow, which may undermine the correlation between the two devices’ readings. Therefore, prior to the mask-to-spirometer (M2S) calibration, we used a second spirometer of the same model to replace the mask, as shown in Figure 5b. This spirometer-to-spirometer calibration quantified the deviation of the flow rate at both ends of the tube as a reference.

**Figure 6:**
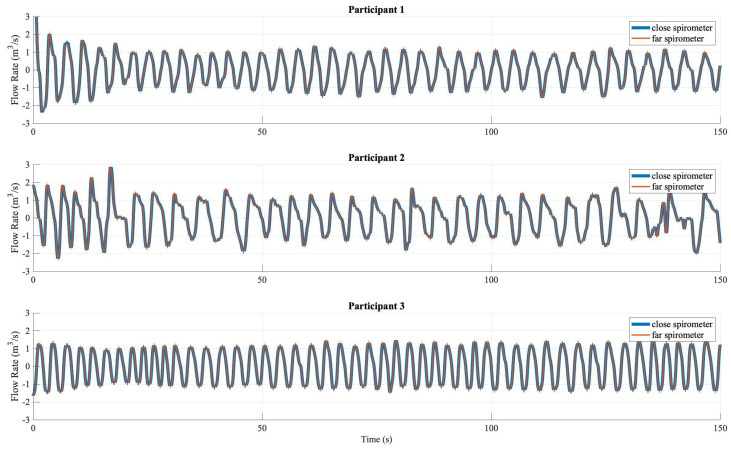
The flow rate data from both spirometers overlapped in the spirometer-to-spirometer (S2S) setup.

**Figure 7:**
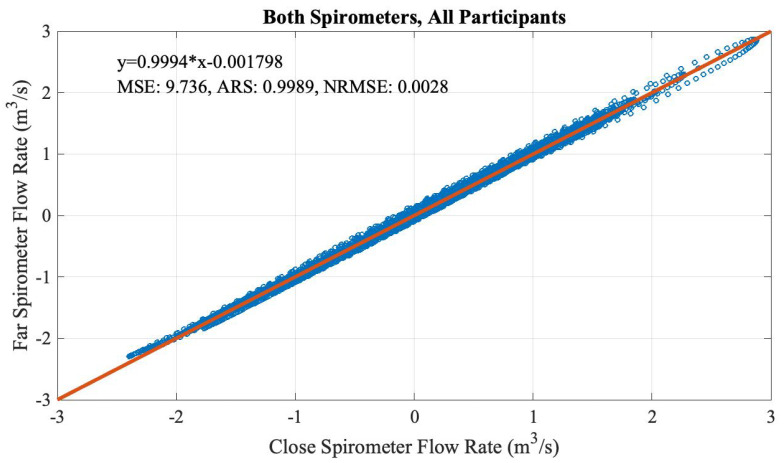
The correlation of the flow rate data from both spirometers in the S2S setting, all participants combined.

Figure 6 shows the time sequences of the flow rate (cubit meter per second) from both spirometers, which overlap almost perfectly. To quantify the correlation, the two flow rates are drawn on different axes in Figure 7. A single term linear approximation was performed using MATLAB’s Curve Fitting Tool (CFTool). From both visually inspecting the data points and the slope value of the fitting function, it could be concluded that the close spirometer was overall slightly more sensitive than the far spirometer, which made sense due to their physical order of encountering the participants’ airway. Furthermore, at the very high negative airflow region, the far spirometer became slightly more sensitive. This may be because during inhaling, the air that came in passed the far spirometer first. This suggested the extra tubing did cause very little friction to the airflow that was distinguishable from the data. However, the difference was negligible on the overall scale and could be explained by the physical position of the two spirometers. Therefore, the M2S setting could be used for deriving the airflow-pressure relationship in our following evaluation.

## 4. Understanding Airflow and Pressure

**Figure 8:**
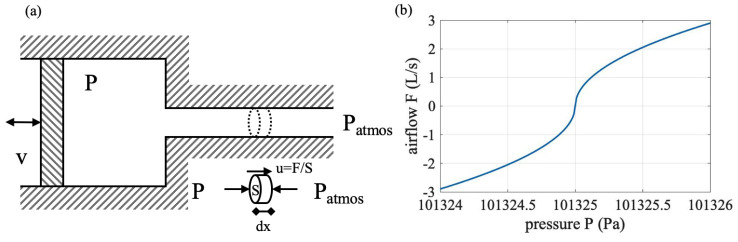
(**a**) An ideal model for the airflow (F)-pressure (P) relationship inside a chamber. (**b**) The theoretical relationship between F and P.

The air pressure inside the mask *P* and the airflow through an orifice can be simplified as a physical model, as shown in Figure 8a, in an ideal situation, that the orifice is through a uniform tunnel with the intersect area of *S*, ignoring any friction. There is a free moving piston that is expanding or shrinking the volume of the chamber, which represents the wearer’s breathing. When *P* is different from the external atmosphere pressure $P_{atmos}$, air will flow at the *F* flow rate until the pressure difference is equalized. The air density is ρ. Take the air from the small part with dx length from the tunnel, and assume the air is flowing with speed u=F/S out of the chamber; the momentum of the air body during a small time period dt is:

(1) $$(P-P_{atmos})\cdot S\cdot dt=\rho \cdot S\cdot dx\cdot du$$

If the air pressure difference is $dP=P-P_{atmos}$, the above equation can be simplified as:

(2) $$dP=\rho \cdot \frac{dx}{dt}\cdot du=\rho \cdot u\cdot du=\frac{1}{2}\rho \cdot du^{2}$$

We have *u* as an integral relationship with *P*:

(3) $$\frac{1}{2}\left(u^{2}-u_{0}^{2}\right)=\int_{P_{atmos}}^{P}\frac{1}{\rho }\cdot dP=\frac{P}{\rho }$$

Bring back u=F/S, and we have:

(4) $$F=S\sqrt{2(P-P_{atmos})/\rho }$$

We can bring in the SI base units, and either side of the equation’s unit is m${}^{3}$/s. Air density ρ is actually dependent on $P_{atmos}$ and the temperature. Here, we assume air density is a constant of 1.225 kg/m${}^{3}$ at standard sea-level, 15 ${}^{{}^{\circ}}$C, $P_{atmos}=$ 101,325 Pa. For the orifice area, we can take the spirometer’s sensing surface of $2.826\times 10^{-3}$ m${}^{2}$. Then, the flow rate (liters/s) can be written as a function of the pressure:

(5) $$F=2.9\sqrt{P-101,325}\;L/s\;\left(P>101,325\right)$$

When the air flows into the chamber:

(6) $$F=-2.9\sqrt{101,325-P}\;L/s\;\left(P<101,325\right)$$

We can plot the function in a realistic flow rate range in Figure 8b. Note that the model is built on the assumption that the pressure inside the chamber is uniformly distributed and the air resistance of the orifice is ignored. The coefficients in Equations (5) and (6) are based on the further condition of standard sea-level at 15 °C and a certain orifice size. Thus, Equations (5) and (6) and Figure 8b only serve to understand the pressure-flow relationship, but not actually measuring the airflow in real-world settings. Both from the equations and the figure, the F−P relationship is clearly non-linear and monotonic, and pressure actually becomes more sensitive in larger flow rate values. To realize converting from pressure to flow rate in our system, we performed experiments with participants and derived the real-world pressure-flow relationship through regression modeling methods.

In [49], dynamic flow analyses were performed during a person’s breathing, speaking, and coughing, which showed the airflow is not uniform in an open face setting in the scale of meters. While the flow analyses in [49] did not analyze the airflow distribution inside the face covering, the study in [50] suggested that the turbulence effect inside a face mask renders the air flow more evenly distributed compared to the open face setting.

This physical model also applies to a larger amount of smaller orifices or pores with varying intersection areas of *s*, such as N95 particulate filters. The only modification would be switching the area *S* to the sum of all pores ∑s in Equation (1). Both will be canceled by each other and removed from the equation, rendering the same resulting equations. The other assumption of this model is that the orifice tunnel does not have friction, which may slow down the air flow speed *u*. This would add additional terms in the F-P relationships in Equations (5) and (6). In the following section, we will take this into consideration in the real-world scenario and overcome the influences from uniformity and friction by mathematical regression from recorded data.

The physical model thus conclusively suggests that the entire airflow between the chamber and the exterior can be calculated from the air pressure inside the chamber. This has a significant implication for the shift of measurement modality: the air pressure can be measured by placing a small sensor in a tiny fraction of the airway, as opposed to that measuring the sum of airflow, requiring sensing elements that cover the entire orifice intersection area.

## 5. Forced Vital Capacity Test

To test the accuracy of our proposed method compared to the medical spirometer devices, we performed the standard forced vital capacity test that is normally conducted during clinical spirometry diagnosis procedures. We followed the instruction from the “Standardisation of Spirometry” [13] to perform the FVC test. The procedure of the FVC test is described in Section 2.1.

### Experiment Procedure

**Figure 9:**
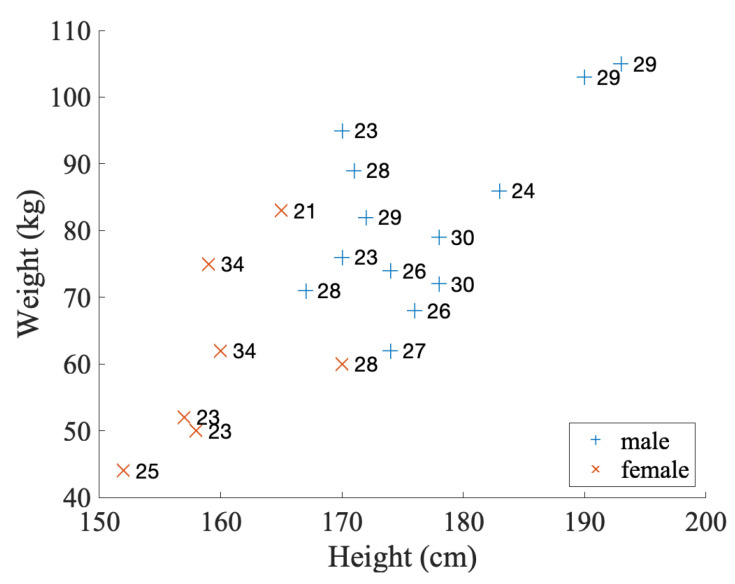
Demographic distribution (the age is written to the right side of every marker).

Twenty participants were recruited aged between 21 and 34. Seven of them were female, and thirteen were male. Their demographic details are shown in Figure 9. The participants were healthy, without any illness symptoms or any known lung-related conditions, e.g., shortness of breath, asthma, etc. The experiment was carried out during the summer months of 2019, and there was no reported seasonal epidemic. Personal and equipment hygiene procedures were carefully observed. Before the recording of every participant, cleaning procedures were carried out for both the participant and the apparatus. The mask was thoroughly disinfected with 70% alcohol. The participant cleaned their mouth with alcohol-based mouthwash and washed their hands and face with soap.

**Figure 10:**
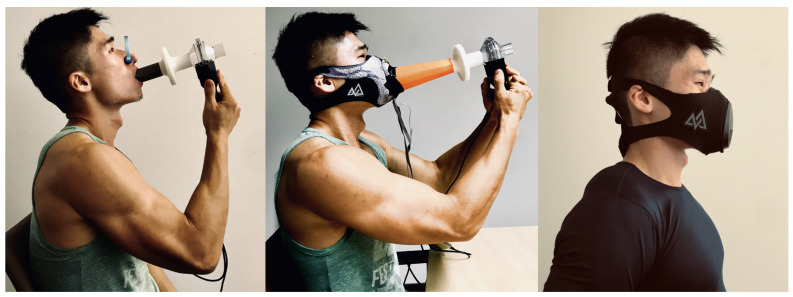
(**Left**) a participant performing a standard clinical forced vital capacity (FVC) test. (**Center**) a participant performing the FVC test with the calibration system. (**Right**) our proposed smart mask functions without extra instrument. Note that our instrument is fully integrated with a standard sports mask routinely used by athletes to control air inflow during exercise without making the mask in any way more obtrusive.

Every participant performed the FVC maneuver 10 times. The M2S setup as described in Section 3.2 and Figure 5a was used. The participants were seated and using the instrumented mask and the spirometer as depicted in Figure 10, center. Between every maneuver, they breathed normally (tidal breathing) for at least five cycles. Approximately one hour of breathing data, containing 200 FVC maneuvers, was recorded for all participants. The participants gave informed consent in accordance with the policies of the University of Kaiserslautern’s Committee for the Protection of Human Subjects, which approved the experimental protocol.

## 6. Signal Processing

**Figure 11:**
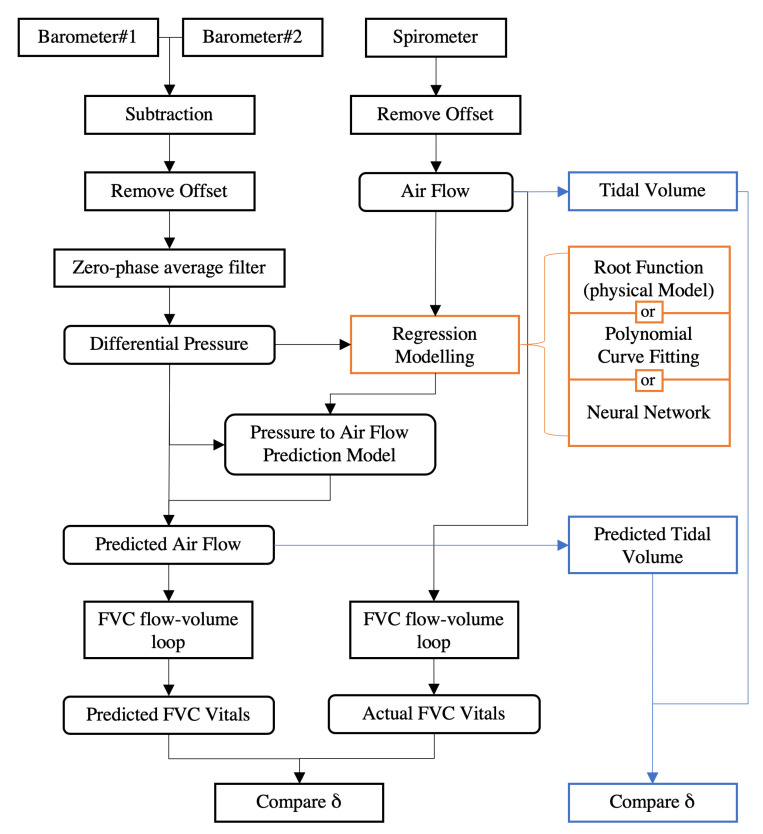
Flowchart of the major evaluation steps.

The overall evaluation methods are illustrated in Figure 11. For the pressure data from every pair of barometers (BMP280#1 and BMP280#2, BMP388#1 and BMP388#2), we calculated the difference from inside the mask to the outside. This differential pressure came with an offset, which was due to the differences during manufacturing. The last half second of the experiment session was used to calculate this offset as the participant had taken off the mask, so that when there was no airflow, the differential pressure was approximately zero. Then, zero phase average filtering with a kernel size of 10 samples was used for smoothing. We also observed offset in the spirometer’s data when there was zero airflow. Therefore, the spirometer’s data were subtracted by the average of the last half second of the recording to remove the offset.

**Figure 12:**
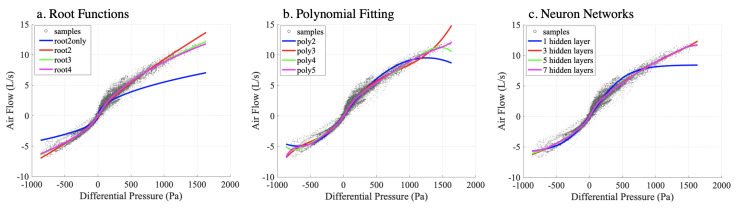
Linear fitting with the root models (**a**), polynomial curves (**b**) and neural network (**c**) regression fitting on the data from all participants combined.

First, we compared the processed data from the differential pressure values measured by the barometers with the airflow values from the spirometer. The values are scatter-plotted in Figure 12 as gray points, as the two measurements at a given sample time represent different physical concepts and have different units. The non-linearity from the measured data showed the strong resemblance of the airflow-pressure relationship from the mathematical model shown in Figure 8. Our first evaluation goal was to see how we could best mathematically interpolate from the barometer’s pressure data to the spirometer’s airflow. We used regression based on the physical model, as well as two other general regression methods: polynomial fitting and neural network.

### 6.1. Physical Model Fitting

Based on Equation (4) from the physical model, we set the differential pressure to *x* and the flow rate to *y* and parametrized the coefficient of the term of $x^{1/2}$. We used regression methods to derive the coefficient. However, since the model was ideal, we added more terms as described in Equations (7) and (8) and refer to them as root functions. A separate fitting function was preformed asymmetrically for positive and negative airflow values.

(7) $$y=a\cdot x+b+c\cdot x^{1/2}+d\cdot x^{1/3}+e\cdot x^{1/4}\;\left(x>0\right)$$

(8) $$y=a\cdot \left(-x\right)+b+c\cdot {\left(-x\right)}^{1/2}+d\cdot {\left(-x\right)}^{1/3}+e\cdot {\left(-x\right)}^{1/4}\;\left(x<0\right)$$

We used linear least squares (LLS) to derive the coefficients of every term. The least absolute residual (LAS) method was used for robustness control. Different root function settings and results are listed in Table 1, from which, “root2only” is the same as Equation (4) with all the coefficients except for *c* set to zero.

From Figure 12, we can observe that as more terms are included in the model equation, the curve aligns closer with the cluster of the sample points.

### 6.2. Polynomial Curve Fitting

Polynomial curve fitting is a common regression technique for non-linear data sample distributions [51]. Polynomial curve fitting assumes a polynomial expression from the observation data (*x*) to the output data (*y*):

(9) $$y=p_{6}\cdot x^{5}+p_{5}\cdot x^{4}+p_{4}\cdot x^{3}+p_{3}\cdot x^{2}+p_{2}\cdot x+p_{1}$$

The coefficients in Equation (9) $p_{1},p_{2},\ldots ,p_{6}$ are determined by the same LLS and LAS methods as with the physical model fitting. We performed from second degree (only with $p_{1},p_{2},p_{3}$) to fifth degree (with all coefficients $p_{1},p_{2},\ldots ,p_{6}$) of polynomial functions with the MATLAB${}^{®}$ Curve Fitting toolbox. Table 2 shows the polynomial coefficients of the all participants inclusive case. Empirically, the higher degree of of the polynomial function is, the more details the regression model can represent from the dataset. In Figure 12, we can observe that as the polynomial degree increases, the curve fits the data cluster better. However, at the range outside the majority of the data clusters, the curve suffers from more fluctuation than the physical model. Furthermore, polynomial functions are not guaranteed to be monotonic. In our case, monotonicity means higher differential pressure will always result in higher airflow. For example, in Figure 12b, we can observe that poly2 and poly4 eventually indicated less airflow as differential pressure reached the boundary of the range of the data.

### 6.3. Neural Network Regression

**Figure 13:**
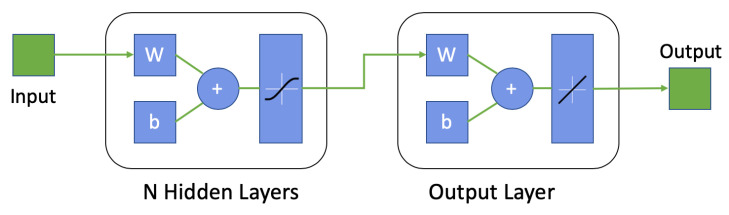
The structure of the neural network regression.

We also evaluated how well an artificial neural network could be used to derive the relationship between the measured pressure and airflow values. The Neural Net Fitting tool from MATLAB${}^{®}$ was used. We used a standard two-layer feed-forward network with sigmoid hidden neurons and linear output neurons, as shown in Figure 13. The network was trained with the Levenberg–Marquardt backpropagation algorithm [52,53]. The data were randomly divided into training-validation-testing by 70%–15%–15% partitioning. The mean squared error of the validation samples was used to automatically stop the training progress. Based on the number of hidden layers *N*, the model was code-named as ANN${}_{N}$ (e.g., one hidden layer was coded as ANN1).

### 6.4. Participant Pool Division Schemes

In our evaluation, the 20 participants were treated according to three separation schemes:

- Individual: A separate model was fitted with the data samples from every participant.
- Inclusive: A single model was fitted with the data sampled from all participants combined.
- Exclusive: The 20 participants were randomly divided into five folds. A separate model was fitted with data from four folds and tested on the remaining fold.

The difference between inclusive and exclusive is that the model is always dealing with a complete stranger in the exclusive scheme. When multiple models were generated from one scheme, we calculated the root mean square of the relevant evaluation parameters.

## 7. Results and Discussion

The goodness of fit (*gof*) is presented as the root mean squared errors (*RMSE*s) between the actual airflow values and the predicted values from the pressure values with the regression models. In general, a more complex model will fit the non-linearity relationship of the data better (i.e., more terms in the root or polynomial functions or more hidden layers in the neural network). However, the benefit becomes trivial after a certain point, and the model may be over-fitted. A smaller RMSE value means the regression model fits better in the data sample cluster. Since the sample cluster did not form a perfectly narrow curve, the RMSE would not be zero.

At this point, we performed the regression with the data sample from all 20 participants combined. The RMSE of all the regression methods are listed in Table 3. From the table, we can see that all three regression models yielded similar fitting results as the model complexity increased.

### 7.1. Predict FVC with Barometers

To see the ability of the barometers in acquiring the useful clinical information needed from spirometry, we used the fitted models to predict the airflow from the pressure measurements. We then constructed the flow-volume loop with the barometer’s data only, as the example shown in Figure 2. From the loop, we then calculated the FVC vitals introduced in Section 5.

To compare the FVC vitals calculated from the two instruments, we define δ as the root mean squared error (RMSE) of the differences divided by the overall range of the vital values from all participants:

(10) $$\delta =\frac{rmse(Vital_{spr}-Vital_{bar})}{max(range\left(Vital_{spr}\right),range\left(Vital_{bar}\right))}$$

**Figure 14:**
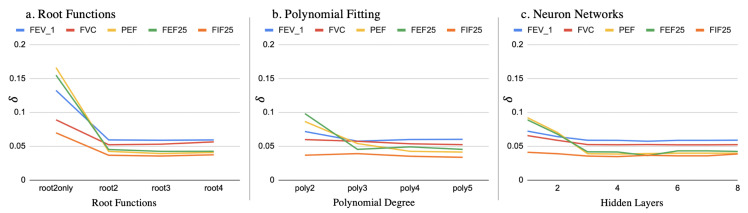
δ of evaluating pulmonary FVC vital parameters between the barometer and the spirometer for the root functions (**a**), polynomial fitting (**b**) and neuron networks (**c**).

Figure 14 shows the δ of the different vitals under exclusive fitting conditions with different models. First of all, we can conclude that the best models could predict all of the FVC vitals with around 5% error when the subject was completely new to the system. Then, we can also observe that the δ values decreased as the model became moderately complex. However, even more complex models bring little improvement over the δ values.

### 7.2. Person Dependency and Customized Fitting

So far, the evaluation has been under the exclusive participant division method, in which case the models did not have any prior knowledge of the participants’ data being tested. However, in the real-world smart wearable scene, a wearable garment such as a face mask can be a private item, which means they can be customized to the specific user or a team of users. Therefore, the inclusive and individual division schemes are also relevant. Table 4 shows the δ values of such cases with the three best performing models. From the table, we can conclude that for all FVC vital parameters, if the system is customized to a single user, the error of prediction can be as low as 2∼3%. Furthermore, if the system is trained with a certain group of users, there is still a slight improvement of the prediction error compared to the exclusive case.

### 7.3. Continuous Tidal Volume Monitoring

A major advantage of such a wearable sensing garment is performing continuous vital sign monitoring. The FVC maneuver is a clinical test procedure that is typically not used during people’s normal breathing activities. We calculated the air volume of all the recorded breathing events, including both the FVC maneuvers and the tidal breathing cycles. Air volume was calculated by integrating the airflow, either measured by the spirometer or interpolated by the model and barometer’s pressure data. To eliminate error accumulation through prolonged integration, a sliding window of 0.5 s with a 10 millisecond window step was used to perform the integration. Figure 3 shows an example of the airflow and calculated air volume from both instruments. From the figure, we can observe that the air volume results from both instruments are on par with each other.

We further calculated the signals’ RMSE-range ratio between the breathing airflow and air volume calculated from both instruments for all participants. It is essentially δ as defined in Equation (10), replacing the vitals that are calculated from complete FVC tests with continuous airflow or air volume data. The results of the best performing models from three regression approaches are presented on the right-hand side of Table 4. With the combination of FVC maneuvers and tidal breathing, further lung volume parameters such as inspiratory reserve volume (IRV), expiratory reserve volume (ERV), inspiratory capacity (IC), etc., can be calculated by selecting part of the respiration volume chart, as illustrated in Figure 3. Therefore, the accuracy of those parameters can be expected to be on the same level as the FVC and TV in Table 4.

From the results, we can first conclude that with all methods, the RMSE between the prediction by the barometer and the spirometer’s measurement was well below 5% of the signal range. When the model was tailored for individual users, the RMSE was as low as 1.5% with the neural network regression method.

### 7.4. The Newer Barometer Version

We compared the higher performance version of the barometer from Bosch-Sensortec, BMP388, with the older version BMP280 in our application to see if the improvement in sensor quality contributed to the spirometry measurement. The key performance improvement aspects of BMP388 over BME280 are:

- operation range of 300 to 1250 hPa over 300 to 1100 hPa,
- barometer noise of 0.03 Pa over 0.2Pa,
- temperature coefficient offset 0.75 Pa/K over 1.5 Pa/K,
- relative accuracy of 8 Pa over 12 Pa,
- output resolution 0.016 Pa over 0.18 Pa,
- one year stability 0.33 hPa over 1 hPa,
- retail price of two Euros over three Euros (as BMP388 lacks the humidity sensor component).

For the purpose of our system, we compared the goodness of fit (*gof*) for the model fitting and the δ for the FVC vitals prediction. With the data from BMP388, the *gof* of the three fitting methods in the inclusive case were: 0.2177 (root4), 0.2182 (poly5), and 0.2188 (ANN7). Compared with the values of the corresponding methods from BME280 in Table 3, 0.1926 (root4), 0.1921 (poly5), and 0.1954 (ANN7), the BMP388 appeared to result in slightly worse regression fitting. We also calculated the δ values as in Table 4 with the BMP388’s measurement. On average, the δ was 0.015 bigger than the values from Table 4, which meant the error margin was 1.5% larger. As the BMP388 was designed to be more sensitive than its predecessors, it is also possible that they are more prone to turbulence, which was also observed and suspected in the study [25]. The bigger margin may also be the result of the microscopic flow unevenness inside the mask chamber.

Thus, we conclude that the newer and more sensitive version of the MEMS barometer BMP388 offers similar performance as the BMP280 sensor, but brings no benefit to wearable spirometry in our system.

### 7.5. Performance and Wearable Prospect

Next, we discuss how our approach can be implemented as a wearable device with real-time measurement. In our evaluation, a USB cable was used to transmit the barometers’ data from the microcontroller to the computer. This was to synchronize the barometers’ data with the reference spirometer as best as possible, as the Vernier SPR-BTA spirometer sends data to the computer with a USB adapter. In [46], a wireless wearable system CoRSA with a single BME280 barometer (instead of a differential pair as in this current study) and the HUZZAH32 microcontroller module, together with other sensors, were already demonstrated. The CoRSA system is powered by a battery and transmits data to a smartphone via Bluetooth. The users can wear the CoRSA system to perform sports activities without hindering their movement freedom. However, the relationship between the single barometer and the actual spirometry-level airflow measurement was not evaluated in [46].

For the computational power, the system essentially goes through three phases in the online operation considering the flowchart in Figure 11:

1. Conditioning the sensor’s raw data through subtraction, removing offset, and filtering, resulting in the differential pressure value.
2. Predicting the air flow from the pressure value using the regression model.
3. Generating results of the pulmonary function test parameters from the flow-volume loop.

Additionally, the regression model should be trained offline with sufficient calibration data. We benchmarked the performance of our method on a 2019, 16-inch Apple${}^{®}$ Macbook Pro with 2.4 GHz 8-Core Intel Core i9 processor, running MATLAB${}^{®}$ 2020a. Our software implementation only utilized a single processor core.

For the three online phases of conditioning, predicting, and generating results, we evaluate all recorded FVC maneuvers. As the FVC maneuvers lasted for different durations (assume *l* seconds), the benchmark results were normalized to a uniform five second period of FVC maneuvers (original benchmark result * 5/*l*). For the offline training, we evaluated the regression fitting process with the entire data recording (approximately one hour of breathing data). Only the most complex models from each model were evaluated as they yielded the least RMSE. The results are listed in Table 5. Note that conditioning and generating results were the same as these two phases were not model-specific.

The benchmark results indicated that the offline training time was less than one second for the root function models and less than 0.1 second for the other models. The signal conditioning and generating the PFT parameters took only 0.0001 s each for raw data segments of five seconds. Once the regression model was trained, the prediction or activation from input barometer data required much less processing power, as the operation was essentially executing Equations (7) and (8), or Equation (9), or activating the neural network depicted in Figure 13. All models could be used to generate flow-rate information and PFT results with less than 0.1 s processing time from raw data of five seconds. Especially with the neural network, the processing time was less than one millisecond.

## 8. Conclusions

### 8.1. Summary

Overall, this work proved that inexpensive and miniaturized barometric pressure sensors can be integrated into a piece of consumer mask apparel to perform accurate spirometry and continuous, transient tidal breathing volume monitoring. The approach is compared with an off-the-shelf certified digital spirometer in a setting where both instruments are connected serially to share the same air flow. A physical model is constructed to derive the theoretical relationship between the pressure inside the mask and the air flow through the orifice, which shows a non-linear square-root relationship under ideal assumptions. An experiment is performed with 20 participants and in total 200 forced vital capacity (FVC) tests, separated by normal tidal breathing to quantify our approach. Three regression approaches are investigated to model the differential pressure-airflow relationship of the real-world data. Using the regression models, we calculate the predicted airflow from the barometers and FVC vital values, which resulted from clinical FVC pulmonary tests. Compared with the clinical spirometer, the error margins for the FVC values from our approach are 5% on average for unknown users and 2∼3% when it is customized to individual users. The error margins for continuous tidal breathing airflow and volume (including TV) are between 1∼3%.

Table 6 compares our approach with other novel spirometer research prototypes mentioned in Section 2.2. Our approach results in the lowest errors while not requiring direct measurement of the airflow through a breathing tube. It is worth recalling that all the novel spirometer studies are developed from the traditional hand-held spirometer structure: a sensing element placed inside a specially-designed breathing tube; while our approach is modifying an off-the-shelf face mask using off-the-shelf and low-cost barometer chips.

### 8.2. Wearable Outlook

The major takeaway from our work is that, with miniaturized sensors integrated in face mask apparel, respiratory activities can be continuously measured on not only the breathing frequency, but also the air volume, with the accuracy on par with clinical pulmonary function test equipment. According to the physical model, the requirement of the garment to enable the spirometry functionality with our approach is only that it forms a chamber with an orifice in front of the wearer’s airway. The model also applies to masks consisting of numerous pores such as particulate filters. From the sensing modality viewpoint, measuring the air pressure requires only a fraction of the airway, and the sensor does not need to be placed in the vent; while traditional spirometers require a sensing mesh to cover the entire airway. Moreover, the mesh-based spirometers are sensitive to humidity, and during operation, a cotton filter is usually needed between the patient and the device to absorb the moisture. On the other hand, the BME280 sensor’s barometer output is independent of the humidity. In fact, BME280 also provides humidity and temperature measurement, as shown in [46]. The form factor of a stand-alone face mask without any extra tubing makes it possible to unobtrusively monitor the user’s tidal breathing continuously while the user performs different activities, as well as dedicated FVC tests. As the sensors are commercially available, they can be easily integrated into personal, fashionable garments, which may appeal to a wider range of consumers.

## Figures and Tables

**Table 1:** Root function coefficients with all participants’ data (inclusive) for Equations (7) and (8).

Model	*a*	*b*	*c*	*d*	*e*
**root2only**	-	-	0.1744	-	-
	-	-	−0.1373	-	-
**root2**	0.0049	−0.3337	0.1466	-	-
	−0.0043	0.1956	−0.1150	-	-
**root3**	0.0001	0.3187	0.5472	−0.8883	-
	−0.0008	−0.1054	−0.3421	0.4707	-
**root4**	−0.0020	−0.0045	0.9210	−3.0140	2.1070
	0.0007	0.0085	−0.5331	1.4570	−0.9274

**Table 2:** Polynomial curve fitting coefficients with all participants’ data (inclusive) for Equation (9).

Model	$p_{1}$	$p_{2}$	$p_{3}$	$p_{4}$	$p_{5}$	$p_{6}$
**poly2**	0.7420	1.5460	−0.0768	-	-	-
**poly3**	0.7747	1.6290	−0.1562	0.0073	-	-
**poly4**	0.7892	1.6550	−0.2128	0.0194	−0.0063	-
**poly5**	−0.0487	0.0207	−0.0001	0.0000	0.0000	0.0000

**Table 3:** Root mean squared error of all fitting methods (inclusive).

Root	RMSE	Polynomial	RMSE	Neural Network	RMSE
**root2only**	0.4237	**Poly2**	0.2152	**ANN1**	0.2371
**root2**	0.2085	**Poly3**	0.1989	**ANN3**	0.2243
**root3**	0.1939	**Poly4**	0.1934	**ANN5**	0.1955
**root4**	0.1926	**Poly5**	0.1921	**ANN7**	0.1954

**Table 4:** δ value of FVC vitals and tidal breathing comparison (the smaller the better). FEV, forced effective volume; PEF, forced peak expiratory flow; FEF, forced expiratory flow; FIF, forced inspiratory flow; TV, tidal volume.

	FVC Vitals					Tidal Breathing	
	FEV1	FVC	PEF	FEF25	FIF25	Airflow	Volume (TV)
**Exclusive (root4)**	0.0591	0.0562	0.0406	0.0423	0.0372	0.0296	0.0285
**Inclusive (root4)**	0.0587	0.0553	0.0388	0.0410	0.0349	0.0249	0.0232
**Individual (root4)**	0.0318	0.0321	0.0241	0.0272	0.0219	0.0196	0.0159
**Exclusive (poly5)**	0.0600	0.0521	0.0411	0.0452	0.0335	0.0362	0.0439
**Inclusive (poly5)**	0.0600	0.0514	0.0378	0.0427	0.0309	0.0246	0.0227
**Individual (poly5)**	0.0303	0.0381	0.0270	0.0274	0.0203	0.0194	0.0154
**Exclusive (ANN7)**	0.0587	0.0518	0.0396	0.0423	0.0362	0.0251	0.0232
**Inclusive (ANN7)**	0.0577	0.0510	0.0369	0.0398	0.0316	0.0243	0.0225
**Individual (ANN7)**	0.0286	0.0327	0.0248	0.0232	0.0224	0.0192	0.0150

**Table 5:** Computational time benchmark results (in seconds).

Model	Conditioning	Predicting	Generating Results	Total Online	Offline Training
**root4**	0.0001	0.0967	0.0001	0.0969	0.7681
**poly5**	0.0001	0.0270	0.0001	0.0272	0.0762
**ANN7**	0.0001	0.0003	0.0001	0.0005	0.0300

**Table 6:** Comparison with state-of-the-art hand-held spirometer studies.

Study	Description	FVC	PEF	FEV1	TV	Flow Range
**Our Approach**	BME280 pair	3.3%	2.5%	2.9%	1.5%	${}^{1}$−7∼12 L/s
**MobiSpiro** [24]	custom MEMS	3%	5%	3%	5%	0∼14 L/s
**FBG** [26]	optical fiber	20%	8%	24%	-	2∼8 L/s
**Open Spirometer** [27]	piezoresistive pressure sensors	${}^{2}$ 6%	-	2%	-	0∼15 L/s
**Mobile Spirometer** [28]	pneumotachometer	${}^{3}$ -	-	-	-	0∼15 L/s
**ATS/ERS** [13,54]	medical society requirement	3%	10%	3%	3%	0∼14 L/s

1: This is the range of our participants’ data. Obviously, a person’s inhale peak flow is less powerful than exhale. However, the barometer’s operation range is at least 100 times more than the range we observed in our experiment. 2: The study in [27] used correlation R as the evaluation measure, and we converted it with 1-R to compare with others. 3: Pulmonary function tests were not performed in the evaluation [28].
